# Neonatal care practice and associated factors among mothers of infants 0–6 months old in North Shewa zone, Oromia region, Ethiopia

**DOI:** 10.1038/s41598-022-14895-3

**Published:** 2022-06-23

**Authors:** Kumera Bekele, Firomsa Bekele, Mathewos Mekonnen, Kemal Jemal, Ginenus Fekadu

**Affiliations:** 1 Department of Nursing, College of Health Science, Selale University, Fiche, Ethiopia; 2 Department of Pharmacy, College of Health Science, Mettu University, Mettu, Ethiopia; 3 School of Pharmacy, Institute of Health Science, Wollega University, P.O Box 395, Nekemte, Ethiopia; 4 School of Pharmacy, Faculty of Medicine, The Chinese University of Hong Kong, Shatin, NT Hong Kong

**Keywords:** Health care, Medical research

## Abstract

Worldwide, the magnitudes of neonatal mortality are estimated to be about 3 million due to insufficient care. The burden of neonatal mortality is high in Ethiopia as compared to high and middle-income countries. The study aimed to assess the neonatal care practice and associated factors among mothers of infants 0–6 months old in Northern Shewa, Ethiopia. A community-based cross-sectional study design was undertaken on a mother living in the North Shewa zone from September 2019 to June 2020. Neonatal care practice was assessed by World Health Organization (WHO) minimum neonatal care package indicators. Over the study period, a total of 245 (62.0%) mothers had a good neonatal care practice. Being urban areas [AOR 5.508, 95% CI 2.170, 13.984], having ANC follow-up [AOR 3.042, 95% CI 1.031, 12.642], lack of adequate information [AOR 0.123, 95% CI 0.054, 0.282] and post-natal care (PNC) [AOR 5.779, 95% CI 2.315, 14.425] were predictors of good neonatal care practice. In our study, there was moderate neonatal care practice among mothers. Therefore, all elements of neonatal care packages should be studied at large.

## Introduction

The time neonate is an essential time for the child to grow and most of them died during their birth^1–4^. Worldwide, the magnitudes of neonatal mortality are estimated to be about 3 million due to insufficient care. Therefore, the mortality rate of under-five children becomes common across different countries^5,6^. Although neonatal mortality rates are also decreasing globally, it was high in Sub-Saharan Africa^7^. Among Sub-Saharan Africa, the rates of mortality were high in Ethiopia^8^. Despite this, the proportion of neonatal death can be prevented by appropriate care of neonates^9^. The components of neonatal care practices should be given during ANC and PNC as per WHO recommendations^10^.

This component includes immunization against tetanus, preparation of mothers for managing complications, ANC follow-up, skilled care of mothers like thermal care, cord care, breastfeeding, and bathing of neonates^10,11^.

In Ethiopia, despite hard work has been tried in the improvement of neonatal care, the status of neonatal care among mothers is still poor. In addition, neonatal care is highly affected by the healthy and knowledge of mothers^11–13^. Home delivery is common in Ethiopia, and ANC and PNC follow-ups are insufficient^14^. This practice of traditional delivery can impact the care of neonates^11,15^. Most inappropriate care occurred during their neonatal period despite the risk of mortality being high in these populations. The magnitudes and determinants of neonatal care had not been studied in the Fiche. As a result, the study aimed to identify the level of neonatal care practice and its determinants.

## Methods

### Study area, design, and period

A Community based cross-sectional study was employed at selected woredas of the North Shewa zone from September 2019 to June 2020. The North Shewa zone has a total population of 1,431,305 from this, about 717,552 are males and 713,753 are females. Fiche is the capital city of the zone, which is found 114 km distant from Addis Ababa.

### Study participants and eligibility criteria

All mothers of infants aged 0–6 months residing at least for 6 months in the catchment area during the data collection period were included in this study while, mothers who were unable to communicate because of seriously ill or impaired cognition were excluded from the study.

### Study variables and outcome measures

The dependent variable was neonatal care practice, which was assessed by WHO core elements. This neonatal care package has 12 that are scored as “Yes” or ‘No’ questions. This element contains immunization from tetanus, preparation of mother to manage complications, antenatal care (ANC), Information on neonatal care, Skilled care at birth, Immediate thermal care, breast-feeding initiation, caring of cord, only breastfeeding of the neonate, washing of newborn, Vaccination during birth, and Infection prevention^10^. The WHO core elements of neonatal danger signs were used to identify the level of knowledge of mothers about neonatal danger signs. Mothers who had mentioned ≥ 3 out of twelve newborn danger signs had good knowledge and those who scored < 3 was classified as poor knowledge^16^.

### Sample size determination and technique

The sample size was calculated by the single population proportion formula whereas, N is the sample size 95% is CI, d is the level of precision, and p is the magnitude of neonatal care practice which is 0.59 (p = 0.59)^17^.

$${\text{N}} = \, \left( {{\text{Z}}\alpha /{2}} \right)^{{2}} {\text{P }}\left( {{1} - {\text{P}}} \right)/{\text{d}}^{{2}} ,$$

$${\text{N}} = \, \left( {{1}.{96}} \right)^{{2}} \left( {0.{59}} \right) \, \left( {0.{41}} \right)/\left( {0.0{5}} \right)^{{2}} ,$$

$${\text{N}} = {368,}$$

where Z = critical value for normal distribution at 95% confidence level which equals to1.96 (Z value at α = 0.05); P = Proportion of neonatal care practice (59.5%**)** from the previous study. d = 0.05 (5% margin of error); and by considering the design effect of 1.5 the sample size becomes 552. After adding the non-response rate of 10% the total sample size was (552 × 10%) + 552 = 607.

A multi-stage clustered sampling technique was used to identify the study participants. First, the zone was stratified to woredas and four woredas were selected by simple random sampling from the total number of woredas. In the second stage kebeles within the selected woreda were selected randomly. Finally, each household in the selected kebeles was selected by systematic random sampling.

### Data collection process and management

A structured questionnaire was developed which was adapted from related literature for neonatal care practices. The tool was developed in English and converted to the local languages ‘Afan Oromo’ to ensure the clarity of questions for the respondents. A pretest was conducted in Bishoftu town out of the study area by taking 5% of our sample size that was not included in the actual study population before the actual data collection takes place. Data were collected by eight (8) B.Sc. nurses and the data collection process was supervised by the principal investigators. Before starting data collection, orientation was given to data collectors for 2 days about the data collection and how to handle the data, and the content of the instrument. The data collectors collected the information through face-to-face interviews of mothers at the house level.

### Data processing and analysis

The data were entered into a computer using EPI-info 3.5.4 software to avoid any errors. Analysis was done using statistical software for social sciences (SPSS) 24.0. Descriptive data were explained as frequency and percentage. Multivariable logistic regression was used to analyze the variable and the variables with a p-value of less than 0.05 were considered a statistically significant association.

### Operational definitions

- *Good neonatal care practices* Those who scored above or equal to the mean score out of 12 items of minimum neonatal care package listed by WHO^10^.
- *Poor neonatal care practices* Those who scored less than the mean scores out of 12 items of minimum neonatal care package listed by WHO^10^.
- *Good knowledge of newborn danger signs* Mothers who had mentioned ≥ 3 out of twelve newborn danger signs^16^.
- *Poor knowledge of newborn danger signs* Mothers who had scored < 3 was classified^16^.

### Ethical approval and informed consent

Ethical clearance was obtained from the ethics review board of Salale University with the reference number SU13/2018. A permission letter was provided to the North Shewa zonal administration office, and in turn to the selected woredas and kebeles in the district, for proceeding with data collection. Informed consent was obtained from all the participants. Additionally, informed consent was obtained from a parent/or legal guardian for participants who are under 18 years of age and have no formal education. To ensure confidentiality, the name and other identifiers of participants and health care professionals were not recorded on the data collection tools. All methods were performed following the relevant guidelines and regulations. The study was performed per the Declaration of Helsinki. The study was registered researchregistry.com with a unique reference number of “researchregistry5882”.

### Consent for publication

Not applicable. No individual person’s personal details, images, or videos are being used in this study.

## Results

### Socio-demographic characteristics of respondents

Of the 607 mothers who were selected to participate, about 395 mothers have completed the interview making the response rate 65.07%. Of the total 395 respondents, 247 (62.5%) were from urban and 148 (37.5%) were from rural areas. About 217 (54.9%) of the mothers were in the age range of 20–29 years. By religion, 279 (70.6%) were Orthodox which was Regarding their educational status, most of the mothers had secondary 119 (30.1%) as their highest educational attainment. Concerning their occupation, 239 (60.5%) mothers were housewives (Table 1).

**Table 1:** Sociodemographic characteristics of respondents in North Shewa zone, Oromia Region, Ethiopia (N = 395).

Variables	Category	Frequency (n)	Percent (%)
Residence	Urban	247	62.5
	Rural	148	37.5
Age of mothers (years)	< 20	35	8.9
	20–29	217	54.9
	30–39	122	30.9
	40–49	21	5.3
Marital status	Married	365	92.4
	Single	10	2.5
	Divorced	14	3.5
	Widowed	6	1.5
Religion	Orthodox	279	70.6
	Protestant	63	15.9
	Muslim	38	9.6
	Others*	15	3.8
Mother’s educational status	No formal education	90	22.8
	Primary	107	27.1
	Secondary	119	30.1
	College and above	79	20.0
Mother’s occupation	Housewife	239	60.5
	Merchants	42	10.6
	Government employee	58	14.7
	Private employee	47	11.9
	Others**	9	2.3
Family monthly income	< 1000	40	10.1
	1000–2000	107	27.1
	2000–3000	40	10.1
	3000–4000	38	9.6
	4000–5000	44	11.1
	> 5000	126	31.9

*Catholic, Waqefana.

**Students, farmers.

### Coverage of maternal and newborn health services

Most of the respondents 363 (91.9%) had ANC follow-up and about 228 (62.8%) received ANC follow-up more than four times, while 19 (5.2%) mothers were received one time only. Most of the women 367 (92.9%) delivered their most recent child in the health facility, while only 28 (7.1%) of women delivered at home. Similarly, in this study 220 (55.7%) of the respondents reported that they get immediate post-natal care (PNC) in their last delivery and most of the mothers 332 (84.1%) did not encounter any difficulties in their last delivery. Out of the total respondents, 303 (76.7%) had birth preparedness for their last delivery (Table 2).

**Table 2:** Utilization of maternal and newborn health services of respondents in North Shewa zone, Oromia Region, Ethiopia (N = 395).

Variables	Category	Frequency (n)	Percent (%)
ANC follow-up in last pregnancy	Yes	363	91.9
	No	32	8.1
The number of ANC follows up	Once	19	5.2
	2–3 times	116	32.0
	More than 3 times	228	62.8
Place of ANC follow-up	Public Hospital	153	42.1
	Health Center	175	48.2
	Health post	35	9.7
TT vaccination in last pregnancy	Yes	332	84.1
	No	63	15.9
Place of last birth	Public Hospital	190	48.1
	Health Center	158	40.0
	Health post	15	3.8
	Private Clinic	4	1.0
	Home	28	7.1
Complication faced during last delivery	Yes	63	15.9
	No	332	84.1
PNC in last delivery	Yes	220	55.7
	No	175	44.3
Birth preparedness for last delivery	Yes	303	76.7
	No	92	23.3

*ANC* antenatal care, *PNC* post-natal care, *TT* tetanus toxoid.

**Figure 1:**
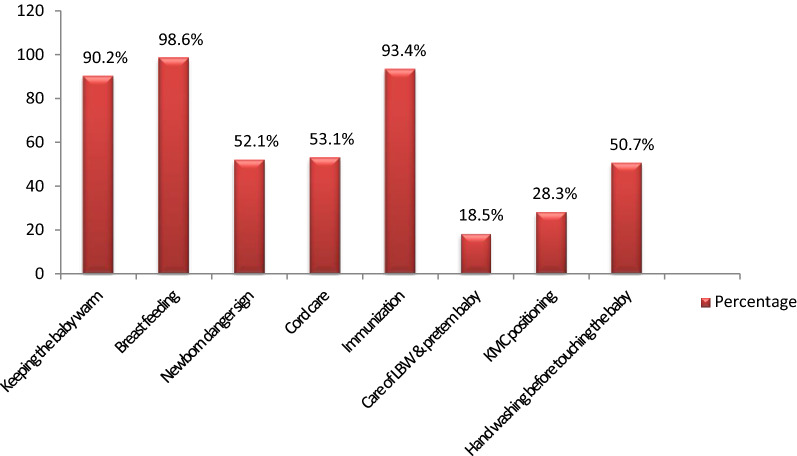
Types of received counseling messages about newborn care before or following last delivery among respondents from Health worker/HEW in North Shewa zone, Oromia Region, Ethiopia (N = 395).

Most mothers 286 (72.4%) had received counseling messages about newborn care before or following their last delivery from a health worker/HEW. The most frequently received counseling messages about newborn care before or following their last delivery were on breastfeeding 282 (98.6%), immunization 267 (93.4%), and keeping the baby warm 258 (90.2%). Fewer women care for the low-birth-weight baby (LBW) & pre-term 53 (18.5%) (Fig. 1). Concerning neonatal danger signs, 204 (51.6%) of mothers have a good knowledge of neonatal danger signs and 191 (48.4%) of mothers had poor knowledge.

### Neonatal care practices

Over the study period, a total of 245 (62.0%) mothers had a good neonatal care practice. Regarding the Newborn thermal care, mothers reported that newborns were dried and/or wiped before delivery of the placenta for 302 (76.5%) of births, while they were wrapped for 364 (92.2%) of births. Of mothers who wrapped their baby before delivery of the placenta, most of them used dry and clean cloths accounted about 188 (47.59%).

Concerning Cord care, a new string or thread was the most used material to tie the cord for 182 (46.1%) of births. On another hand in most of the respondents 16 (4.05%), the blade was commonly used to cut the cord after delivery of their recent baby while in 7 (1.77%) of mothers’ the scissor was used to cut the cord.

Regarding their breastfeeding, out of total respondents, only 204 (51.6%) mothers reported that their newborns were breastfed within the first hour after delivery,73 (18.5%) between 1 and 6 h, 62 (15.7%) between 6 and 24 h, 56 (14.2%) were after 24 h. About 258 (65.3%) mothers reported that they bathed their newborn child for the first time after 24 h of birth. Of the total respondents’ most of the mothers 269 (68.1%) reported that their newborns were given vaccination (BCG and Polio-0) on their first day of birth (Table 3).

**Table 3:** Immediate neonatal care practices of mothers in North Shewa zone, Oromia Region, Ethiopia (N = 395).

Variables	Category	Frequency	Percent (%)
Condition of the cloth which was used for wrapping the bay	Clean cloth and new	144	36.46
	Clean and dry	188	47.59
	Wet cloth	63	15.95
Placement of new-born immediately after delivery	On the floor	8	2.0
	On the mother’s chest/abdomen	245	62.0
	Beside the mother	42	10.6
	With someone else	47	11.9
	On new-born bed/table	53	13.4
Instruments used to cut the cord	Blade	16	4.05
	Knife	5	1.27
	Scissor	7	1.77
The material used to tie the cord	String/thread	182	46.1
	Fiber from interpolant	62	15.7
	Cord not tied	12	3.0
	Do not know	137	34.7
	Other*	2	0.5
Applications to the cord immediately after cutting	Oil applied	4	5.2
	Butter applied	37	48.1
	Ash applied	5	6.4
	Ointment/powder applied	25	32.5
	Other substance applied**	6	7.8
First milk /colostrum that came from breast given for the new-born baby	Yes	291	73.7
	No	104	26.3
New-borns have given something other than breast milk during the first 3 days	Yes	118	29.9
	No	277	70.1
Types of foods/liquids given for new-borns to feed during the first 3 days	Plain water	97	82.2
	Sugar water	35	29.7
	Milk (other than breast milk)	89	75.4
	Fresh butter	28	23.7
Bathing	Immediately after delivery	54	13.7
	Within 24 h after birth	83	21
	After 24 h of birth	258	65.3

*BCG* Bacille Calmette-Guérin, *OPV0* Oral polio vaccine at birth.

*Cotton tie.

**Chlorhexidine, Herbs.

### Factors associated with neonatal care practices among mothers

Residence of mother, educational status of the mother, mother’s occupation, husband’s educational status, family monthly income, ANC follow up, number of ANC follow-up, information on neonatal care, birth preparedness, and complication readiness, having PNC service and maternal knowledge on neonatal danger sign were factors associated with neonatal care practice among mothers on bivariate analysis.

In multiple logistic regressions, mothers who lived in urban areas were 5.5 times more likely to have good neonatal care practices when compared to mothers who lived in rural areas. [AOR 5.508, 95% CI (2.170, 13.984)]. Mothers who had ANC follow-up in their last pregnancy were 3 times more likely to have good neonatal care than mothers who did not attend ANC follow-up [AOR 3.042, 95% CI (1.031, 12.642)].

Mothers who did not get adequate information from a health worker or HEW on neonatal care before or following their delivery were 0.12 less likely to have good neonatal care practices as compared to mothers who had to get adequate information on the newborn issues [AOR 0.123, 95% CI (0.054, 0.282)].

A mother who had birth preparedness & complication readiness in their last pregnancy was 5.3 times more likely to have a good neonatal care practice as compared to others [AOR 5.311, 95% CI (2.055, 13.723)] (Table 4).

**Table 4:** Multivariate logistic regression analysis result of factors associated with neonatal care practice among mothers of infants 0–6 months in North shewa zone, Oromia region, Ethiopia (N = 395).

Variables	Category	Neonatal care practice		COR (95% CI)	AOR (95% CI)	p-value
		Poor	Good			
Residence	Rural	100 (66.67%)	148 (42.90%)	1	1	< 0.001*
	Urban	50 (33.33%)	197 (57.10%)	2.66 (1.164–3.047)	5.508 (2.170–13.984)	
Mothers’ education status	No formal education	68 (45.33%)	22 (8.98%)	1	1	0.531
	Primary	46 (30.67%)	61 (24.90%)	4.10 (1.008–7.061)	2.236 (0.021–2.649)	0.242
	Secondary	31 (20.67%)	88 (35.92%)	2.140 (1.034–6.240)	1.139 (0.015–1.258)	0.079
	College and above	5 (3.33%)	74 (30.20%)	5.214 (2.071–8.518)	3.639 (0.086–4.733)	0.661
Mothers occupation	Housewife	126 (84%)	113 (42.48%)	1	1	0.257
	Merchants	8 (5.33%)	34 (12.78%)	4.74 (0.110–6.835)	3.580 (0.260–9.234)	0.340
	Government employee	2 (1.33%)	56 (21.05%)	6.59 (0.435–10.374)	4.997 (0.286–7.431)	0.271
	Private employee	11 (7.33%)	36 (13.53%)	0.12 (0.094–1.134)	0.92 (0.061–6.08)	0.711
	Others	3 (2.00%)	6 (2.26%)	0.61 (0.350–7.646)	0.83 (0 0.076–9.091)	0.895
Family monthly income (Ethiopian birr)	< 1000	32 (21.33%)	8 (3.27%)	1	1	0.237
	1000–2000	76 (50.67%)	31 (12.65%)	1.63 (1.01–3.081)	1.28 (0.051–1.533)	0.142
	2000–3000	10 (6.67%)	30 (29.41%)	7.35 (2.025–10.102)	6.30 (0.092–8.984)	0.067
	3000–4000	5 (3.33%)	33 (32.35%)	2.20 (1.152–4.93)	1.72 (0.173–2.987)	0.650
	4000–5000	13 (8.67%)	31 (12.65%)	0.36 (0.28–2.460)	0.39 (0.284–6.854)	0.683
	> 5000	14 (9.33%)	112 (45.71%)	3.35 (1.13–6.700)	1.09 (0.267–4.45)	0.904
ANC follow-up	No	25 (11.63%)	7 (3.89%)	1	1	0.002*
	Yes	190 (88.37%)	173 (96.11%)	3.25 (1.140–6.23)	3.042 (1.031–12.642)	
Information on neonatal care	Adequate	27 (18%)	197 (80.41%)	1	1	< 0.001*
	Inadequate	123 (82%)	48 (19.59%)	0.053 (0.032–0.090)	0.123 (0.054–0.282)	
BPCR	No	76 (50.67%)	22 (8.98%)	1	1	0.001*
	Yes	74 (49.33%)	223 (91.02%)	10.410 (6.052–17.909)	5.311 (2.055–13.723)	
Knowledge of neonatal danger sign	Poor	116 (77.33%)	75 (30.61%)	1	1	0.645
	Good	34 (22.77%)	170 (69.39%)	7.733 (4.838–12.360)	1.331 (0.394–4.492)	

*CI* confidence interval, *COR* crude odd ratio, *AOR* adjusted odd ratio.

*p-value < 0.05.

## Discussion

Neonatal care and their health can be improved by a multidisciplinary approach in Ethiopia^14^. The magnitude of neonatal care practice in our study area was 62.0%, which was almost like the previous study done in southwest Ethiopia (59.5%)^17^, but it was relatively higher than the study conducted in Addis Ababa, Ethiopia (29%)^18^.

Most of the elements of the neonatal care practice along the continuum of care were somewhat good. This finding was almost higher than a previous study done in southwest Ethiopia^17^. This may be described relatively by a good knowledge of mothers regarding neonatal health and neonatal care that increases its utilization.

Neonatal care practice was higher among mothers from the urban area as compared to their counterparts. Mothers who lived in urban areas were 5.5 times more likely to have good neonatal care practices when compared to mothers who lived in rural areas [AOR 5.508, 95% CI (2.170, 13.984)]. This was consistent with other prior studies conducted in Southwest Ethiopia^17,19,20^. This is due to the ease of access to information for mothers from urban areas.

Similarly, this study revealed that mothers who had ANC follow-up in their last pregnancy were 3 times more likely to have good neonatal care than their counterparts [AOR 3.042, 95% CI (1.031, 12.642)]. This was also consistent with other prior studies done in Nepal^21^. This is due to ANC follow-up which insights into mothers’ neonatal care practice.

Mothers who did not get adequate information from a health worker or HEW on neonatal care before or following their delivery were 88% less likely to have good neonatal care practices as compared to mothers who had to get adequate information [AOR 0.123, 95% CI (0.054, 0.282)]. This was almost consistent with other prior studies done in Nepal^21^, and East Gojjam, Ethiopia^22^. This may be due to the reason that getting adequate information on neonatal care and other newborn issues is very important for mothers to increase their awareness and practices toward their newborn care.

This study revealed that mothers who had birth preparedness and complication readiness were 5.3 times more likely to have good neonatal care practices as compared to others [AOR 5.311, 95% CI (2.055, 13.723)]. Similarly, mothers who got immediate PNC services were 5.8 times more likely to have good neonatal care practice as compared to others [AOR 5.779, 95% CI (2.315, 14.425)]. This finding was almost like other prior studies done in Nepal^21^. This is due to the PNC services of mothers, as they are expected to get essential information and be counseled about every aspect of newborn issues which helps them to have good practices toward their newborns.

### Strength and limitations of the study

As the strength, the determinants of neonatal care practice at both the community and individual levels were studied. As a limitation since the study was cross-sectional it may not be strong enough to demonstrate a causal relationship between dependent and independent variables due to the nature of the study. Similar studies were done on neonatal care practice and associated factors among mothers are limited in our country to make comparative discussion. In addition, neonatal care practices were determined based on mothers’ reports, which might have been forgotten. Therefore, precaution should be given while interpreting the findings.

## Conclusion

The study showed that almost there was moderate neonatal care practice among mothers in the study area. Besides this, the big difference was founded in the coverage of the components of the neonatal care package. Place of residence, attending ANC follow-up, receiving information/counseling before, or following delivery from a health worker or HEW, and birth preparedness and complication readiness (BPCR) were found to be determinants of neonatal care practice. Therefore, Policymakers and healthcare providers need to create awareness in the community to improve the coverage of the neonatal care package components before, during, and after birth. Community-based programs are expected to address the poor practices to improve neonatal outcomes. Besides this, improving antenatal care follow-up, and family planning service is recommended to assure good newborn care practices among mothers. Furthermore, it is recommended to conduct qualitative research to explore the reasons and beliefs associated with poor neonatal care practices.

## Data Availability

The dataset of this article is accessible on reasonable request from the corresponding author.
